# Vision beyond the Field-of-View: A Collaborative Perception System to Improve Safety of Intelligent Cyber-Physical Systems

**DOI:** 10.3390/s22176610

**Published:** 2022-09-01

**Authors:** Manzoor Hussain, Nazakat Ali, Jang-Eui Hong

**Affiliations:** 1 Department of Computer Science, Chungbuk National University, Cheongju 28644, Korea; 2 School of Innovation, Design, and Engineering, Malardalen University, 72220 Vasteras, Sweden

**Keywords:** intelligent cyber-physical systems, autonomous driving systems, collaborative perception, safety, logistic chaos map-based encryption

## Abstract

Cyber-physical systems (CPSs) that interact with each other to achieve common goals are known as collaborative CPSs. Collaborative CPSs can achieve complex goals that individual CPSs cannot achieve on their own. One of the examples of collaborative CPSs is the vehicular cyber-physical systems (VCPSs), which integrate computing and physical resources to interact with each other to improve traffic safety, situational awareness, and efficiency. The perception system of individual VCPS has limitations on its coverage and detection accuracy. For example, the autonomous vehicle’s sensor cannot detect occluded objects and obstacles beyond its field of view. The VCPS can combine its own data with other collaborative VCPSs to enhance perception, situational awareness, accuracy, and traffic safety. This paper proposes a collaborative perception system to detect occluded objects through the camera sensor’s image fusion and stitching technique. The proposed collaborative perception system combines the perception of surrounding autonomous driving systems (ADSs) that extends the detection range beyond the field of view. We also applied logistic chaos map-based encryption in our collaborative perception system in order to avoid the phantom information shared by malicious vehicles and improve safety in collaboration. It can provide the real-time perception of occluded objects, enabling safer control of ADSs. The proposed collaborative perception can detect occluded objects and obstacles beyond the field of view that individual VCPS perception systems cannot detect, improving the safety of ADSs. We investigated the effectiveness of collaborative perception and its contribution toward extended situational awareness on the road in the simulation environment. Our simulation results showed that the average detection rate of proposed perception systems was 45.4% more than the perception system of an individual ADS. The safety analysis showed that the response time was increased up to 1 s, and the average safety distance was increased to 1.2 m when the ADSs were using collaborative perception compared to those scenarios in which the ADSs were not using collaborative perception.

## 1. Introduction

Cyber-physical systems (CPSs) integrate sensing, computational resources, control mechanisms, and networking resources into the physical systems, connecting them to interact with each other. The advancements in CPSs have the potential to make the systems more responsive, precise, efficient, and reliable. CPSs have brought a revolution in intelligent systems, e.g., from autonomous driving systems to smart grids and health care to smart manufacturing systems. The collaboration of CPSs provides functionalities beyond the capabilities of individual systems. Intelligent vehicular cyber-physical systems (VCPSs) can be considered as the typical example of collaborative CPSs that integrate the vehicular cyber world with the vehicular physical world. Integrating computing and physical resources in VCPSs enables autonomous driving systems (ADSs) to interact with each other and their surrounding infrastructure (i.e., roadside units) to improve traffic safety, robustness, efficiency, and reliability.

ADSs in VCPSs have the potential to process traffic information locally by communicating with other ADSs through collaboration. The VCPSs provide many benefits in terms of perceptual robustness that enables the reduction in traffic congestion and increases traffic safety. VCPSs mostly rely upon onboard sensors such as cameras and LiDAR to perceive the surrounding environment. Despite significant advancements in sensor technology, the onboard sensors’ capability is bounded by their range and field of view. Additionally, occluded and out-of-sight objects such as pedestrians and pets, as well as other moving objects, impose challenges on perception systems. Lack of situational awareness regarding occluded objects or objects beyond the range of sensors can cause catastrophic safety concerns for ADSs.

The perception of individual ADSs has its limitations [1,2]. For example, the capability of perceiving surrounding objects by the onboard sensors can be limited when perceiving occluded objects. Therefore, the collaboration among ADSs can increase their capabilities to perceive surrounding objects by exchanging information through vehicular communication, enabling the ADSs to detect objects that were not detected by their own onboard sensors [3]. In collaborative perception, the perception ranges of ADSs can be extended beyond the line-of-sight and field-of-view; hence by eliminating the blind spots, occluded objects can be perceived.

Different sensing fusion techniques are used to develop collaborative perception systems in VCPSs. The commonly known fusion techniques are the homogeneous sensor data fusion of multiple ADSs, heterogeneous sensors’ fusion of individual ADSs, and sensor data fusion of heterogenous sensors of multiple vehicles [4,5]. However, our approach focuses on the homogenous sensor (i.e., camera sensor data) data fusion technique of multiple ADSs.

Camera image stitching is a widely used technique in real-life applications such as ADSs, virtual reality, aerospace, and medical imaging [6,7]. It is used to enhance the field of view by combining multiple overlapping local fields of view. The advance in VCPSs has enabled the ADSs to share their perception with other ADSs through vehicle to vehicle (V2V) communication. Therefore, in the ADSs domain, image stitching can combine the individual perception of ADSs to increase the sensor’s field of view. Generally, image stitching techniques can be divided into two types: pixel-based stitching and feature-based stitching. The pixel-based stitching method compares the intensity of each pixel in the image and reduces the difference between the pixels to perform stitching [8]. However, the feature-based stitching [9] technique first extracts the features (i.e., points, corners, or shapes) from the images, and then the correspondence between the extracted features is established.

### 1.1. Motivation

**Figure 1:**
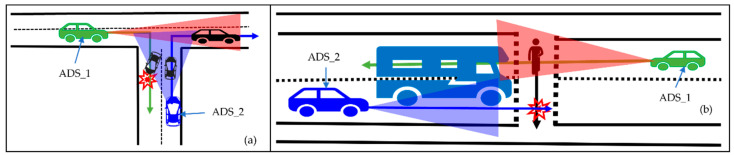
Occlusion in a real traffic scenario. (**a**) represents occlusion on T-junction, and (**b**) represents a hazardous scenario caused by occlusion.

Just as with other components of ADSs, sensors are susceptible to failure and have limitations on frequency, resolution, and field-of-view [10]. Therefore, relying on a single input data to make control decisions for ADSs may create safety issues. The perception system of individual ADS gets worse while detecting occluded objects. Figure 1 shows examples of occlusion in real traffic scenarios. The future trajectory of ADS_1 is shown in the green arrow, and the future trajectory of ADS_2 is shown in the blue arrow. From Figure 1a, it can be observed that the individual perception system of each vehicle has its own limitation on sensing range and field-of-view. For example, in Figure 1a, the broken vehicle is occluded to ADS_1 at T-junction. Hence, the ADS_1 perception system cannot detect the broken vehicle. This is the same as how the black vehicle (i.e., which is not in the sensing range of ADS_2) is occluded to ADS_2. The purpose of detecting beyond the vision or occluded object is to enhance the situational awareness of ADSs in VCPSs, improving traffic safety by better planning their future trajectory.

Earlier detection of the objects through collaboration can help the ADS to make better decisions for the future trajectory. Figure 1a shows that ADS_2 can detect the broken vehicle that is occluded to ADS_1. In collaborative perception, for example, the ADS_2 shares its perception with ADS_1 so that the ADS_1 can detect the broken vehicle in advance and perform better planning to avoid traffic congestion and safety-related issues.

Similarly, consider a traffic scenario where the traffic light was green for pedestrians, as shown in Figure 1b. However, a pedestrian was crossing the road, and in the middle of the road, the traffic light turned green for ADS_2 and the bus. Due to occlusion by the bus, the ADS_2 could not detect the pedestrian behind the bus, resulting in the possibility of ADS_2 hitting the pedestrian despite applying the brake. However, this safety-related issue could be solved through collaborative perception, where both ADSs share their perception. Figure 1b shows that the ADS_1 can detect the pedestrian. Therefore, sharing the perception of ADS_1 to ADS_2 can extend the sensing range, improving traffic safety.

However, a malicious vehicle may send phantom information in collaborative perception, and this may cause threats to the safety of participating ADSs. This issue can be solved by encrypting each image frame shared by ADSs so that phantom information can be avoided.

Various object detection and segmentation models have been proposed in the recent literature [11,12,13,14]. Modern object detection techniques based on camera images [15,16] and LiDAR data [17,18] use different deep learning algorithms, such as convolutional neural networks (CNN) [19], to process data and region proposal networks (RPN) to detect the object. Many research studies have been reported on collaborative perception focusing on improving the individual ADS’ precision [20,21,22]. Previous studies mainly focused on research issues in collaborative perception, such as improving the precision of individual ADSs, the impact of data exchange on the network, the format of data to be exchanged, data fusion at the edge, and fog computing. However, the safety aspects of collaborative perception have not been fully explored. Both camera and LiDAR sensor data are used to detect objects in ADSs. Although the LiDAR sensor has better precision in 3D space it is computationally very expensive compared to camera sensors’ data, because a typical LiDAR frame contains up to 100,000 LiDAR points, and the size of each frame is almost 4 MB [10]. Exchanging such a massive amount of data would be computationally very expensive. In contrast to the LiDAR frame, the compressed camera sensor data size is very low. Therefore, we opted to use camera sensor data for our collaborative perception systems and investigated the safety aspects of collaborative perception.

### 1.2. Main Contributions

This paper proposes a collaborative perception system that enables ADSs to detect occluded objects and objects beyond the field of view. It also enhances the object detection rate. Our proposed approach uses the camera sensors’ image fusion and stitching techniques. The camera sensor data from multiple ADSs are fused to make a collaborative perception system that realizes end-to-end object detection to improve situational awareness. The compression technique used in our approach enables the removal of the computational burden from the network. It only takes a few milliseconds (i.e., 301 ms) to transmit data from one ADS to another, which realizes real-time object detection feasible in both in-vehicle and fog computing environments. We believe that our computationally less-expensive collaborative perception system can be deployed on in-vehicle as well as fog computing systems. Our paper makes the following contributions:

**Technique:**

- A collaborative perception system enables VCPSs to detect occluded objects based on the camera sensor image fusion and stitching technique, improving the safety of VCPSs.
- A real-time collaborative object detection system is proposed using the faster region-based convolutional neural network (Faster R-CNN) to detect and localize objects in collaborative perception.
- A logistic chaos map-based real-time end-to-end encryption technique to encrypt image frames in collaborative perception in order to avoid phantom information shared by malicious vehicles.

**Dataset:**

- We generated a dataset containing 1895 labeled simulation-based images that can be used for object detection in real-time in the CARLA simulator [23]. It can also be used to evaluate the performance of the real-time object detection algorithm in collaborative perception systems. The dataset contains four classes: *walker*, *passenger_car*, *truck*, *motorcycle*, and *cycle*.

**Evaluation:**

- Empirical analysis shows that the collaborative perception in our proposed approach enhanced the situational awareness of ADSs and outperformed the individual perception system of ADSs in terms of detecting occluded objects and enhancing safety.

The rest of the paper is organized as follows: Section 2 presents the related work, while the system model and the proposed approach are presented in Section 3. Section 4 describes the experimental setup, and performance evaluation is presented in Section 5. Finally, Section 6 concludes this paper.

## 2. Related Work

*SIFT-based Feature Extraction and Image Fusion:* the SIFT algorithm [24] is a widely used computer vision algorithm to detect and describe features in images. The SIFT algorithm first searches for the feature points in spatial scale-space and then extracts the scales, rotation invariants, and position of the feature points. The next step is to combine the matching points, eliminate the mismatched features, and fuse both images. The work by [24] exploits a dense SIFT descriptor for ghost-free image fusion. The authors used the SIFT algorithm to extract contrast information from the source image for fusion. In another study [25], SIFT was used to extract features and feature matching for the image fusion of different vehicles. The authors proposed a co-operative visual augmentation algorithm based on inter-vehicle image fusion for autonomous driving systems. It was used for feature selection and description. Another study used the SIFT algorithm to stitch the unmanned aerial vehicle (UAV) low-altitude remote sensing images [26]. In the study of authors [27], the SIFT algorithm was used as a feature extractor for image stitching. The image stitching in this work was applied to enhance the UAV navigation in GNSS-denied regions. The work by the authors [28] used the SIFT algorithm to stitch video image frames in real time. Both studies used the SIFT algorithm as a feature extractor and feature matching algorithms to stitch video image frames. The studies of the authors in [7,29] exploited the application of the SIFT algorithm in the autonomous driving domain for stitching the image frames from multiple autonomous vehicles. They proposed multi-scene image stitching methods based on SIFT.

*Collaborative Perception Systems:* collaborative perception based on data fusion can be achieved using three different methods: high-level, low-level, and feature-level. Data fusion techniques are actively used in object detection and tracking to improve accuracy and precision. The authors used data fusion for better detection and tracking of the object in autonomous driving systems [30]. The research work by [20] exploits the high-level fusion methods on multi-sensors for 3D object detection. The proposed approach was used to detect and track dynamic objects through the fusion of multiple sensors. The authors in [31] used a high-level sensor data fusion technique for co-operative perception systems. They proposed an architecture named Car2X-based perception based on high-level sensor data fusion. The research work by [10] proposed a point cloud-based co-operative perception framework for connected vehicles to achieve high precision in object detection. The proposed framework uses the feature-level fusion technique to achieve the co-operative perception of multiple vehicles. The proposed framework achieves faster edge computing with low communication delay and computing cost, and it only requires 71 ms for feature selection. In another study, the same authors proposed a co-operative perception called Cooper [32], based on the raw LiDAR data fusion from multiple vehicles, to improve object detection precision. The proposed method was based on a low-level data fusion technique that significantly improved detection performance. 

To the best of our knowledge, no existing works have been reported to implement the concept of the homogeneous sensor (i.e., camera sensor) data fusion of multiple ADSs for collaborative perception. Our contribution toward collaborative perception differs from the existing approach [33] regarding sensor type, positions, and data used for fusion methods. The authors used LiDAR point cloud data for fusion. Their sensor placement differs from our approach as the sensors are fixed to a specific location, such as roundabouts and T-junctions. The objects beyond the fixed sensors’ range in specific locations are still undetected. For example, their approach used fixed sensor sets for T-junction and roundabouts scenarios. However, those objects beyond these driving scenarios are still undetected.

In contrast to the aforementioned approach, we use the camera sensors’ image frames mounted on the ADSs irrespective of fixed locations, eliminating blind spots anywhere in the driving scenarios. We also compare the fusion scheme with F-Cooper and Cooper and [33] in terms of data used for fusion. All the above-mentioned approaches used LiDAR points to clouds for fusion. On the other hand, we use the camera sensor’s image frames for collaborative perception. We also investigated the safety aspects of collaborative perception.

## 3. System Model and Proposed Approach

### 3.1. System Model

**Figure 2:**
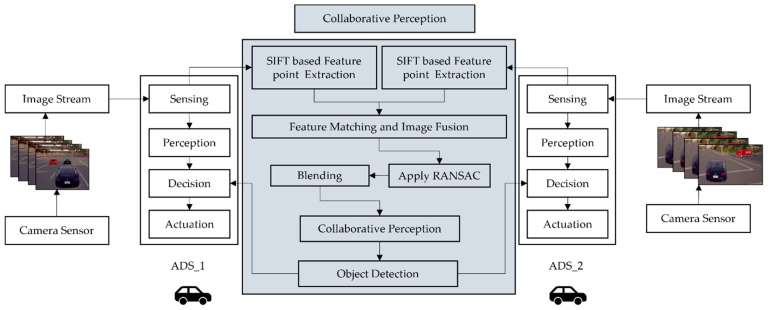
A proposed approach for collaborative perception.

The proposed collaborative perception system considers homogenous sensors, i.e., camera image fusion and stitching of multiple ADSs in VCPSs, as shown in Figure 2. Each ADS can sense through the camera sensor and process the camera images at its local processor. The camera sensor’s images are shared with nearby ADSs through VCPS-enabled V2V communication in the collaborative perception system. Our system assumes that each camera sensor mounted on ADSs was well-calibrated and captured images are compressed before being transferred. Note that the collaborative perception system is not responsible for controlling ADSs directly. The ADSs will use the collaborative perception system’s information as well as its own local perception system to make control decisions. The role of the collaborative perception system is to assist the ADSs in making safer control decisions to avoid safety-threatening scenarios such as potential hazards caused by occluded objects. The broad aspects of network delay, communication loss, and security protocol for cyber-attack are beyond the scope of this paper. However, the end-to-end encryption technique is used to encrypt the image frames while sharing each image frame from one ADS to another.

In our system, the camera sensors capture the surrounding environment, and the local perception system of ADSs understands the scenes, and detects and localizes the objects. However, in the case of occlusion or objects beyond the field of view, the local perception cannot detect and localize those objects. Using image fusion and stitching techniques, our collaborative perception system enables the participating ADSs in collaborative perception to increase their situational awareness by detecting and localizing the objects beyond their field of view.

### 3.2. Image Fusion and Stitching-Based Collaborative Perception System

Video image stitching has been gaining popularity due to its application in diverse domains, such as virtual reality, unmanned aerial vehicle (UAVs) surveillance, and ADSs [34,35]. Just as with image stitching, in the video stitching algorithm, the individual frames of the video stream are stitched in real-time. Different algorithms are used in the video stitching method to find the feature points in each image frame. After feature points are detected, the random sample consensus (RANSAC) algorithm is used to generate the homograph matrices for each image frame in the spatial and temporal domains. The linear blending is applied to the overlapping region of the video image frame for stitching and to get the panoramic view.

Many feature extractors and keypoint detectors have been proposed, such as oriented fast rotated brief (ORB) [9], speeded up robust features (SURF) [36], and binary robust invariant scalable keypoint (BRISK) [37]. However, compared to these stitching and feature extraction techniques, scale invariant feature transform (SIFT) [38] is more robust for image transition, scale, illumination, and angle changes, more efficient when processing them, and easier to implement. Due to these characteristics, SIFT is the most used feature extractor and image fusion algorithm. In order to extract the features for image fusion and stitch the image frames, we used the SIFT algorithm. This algorithm is used as a local keypoint detector and descriptor. The SIFT algorithm extracts the features of objects in the scene based on different scales, rotations, the geometric transformation of the object, and illumination. It has good robustness to angle change and noise affine transformation, which is crucial in the ADSs domain.

The SIFT algorithm first describes the features of the image frame by finding feature points and related descriptors in an image. The next step is to extract the feature points of the input image and determine the positions and orientation of the feature points. The feature vector of the SIFT algorithm uses the nearest neighbor Euclidean (NNE) distance between the feature points. Based on the NNE distance, the feature points can be aligned. After calculating the distance between the feature points, the images are fused. However, the SIFT algorithm detects many feature points; these features are sometimes mismatched. Therefore, the RANSAC algorithm [39] is applied to remove unnecessary and mismatched feature points. The RANSAC algorithm also removes the misaligned feature points, produces a seamless panorama of the stitched image, and removes the ghosting artifact. The primary purpose of applying image blending is to produce the fused image where no transition can be observed between the original images and the fused image (i.e., stitched image). Finally, Faster R-CNN is applied to detect and localize the object in collaborative perception. In the following subsections, we explain each step involved in SIFT algorithm-based collaborative perception systems, including SIFT-based feature extraction, feature mapping-based image fusion, RANSAC application to remove ghosting artifacts, mismatched features and blending, and finally, the Faster R-CNN-based collaborative object detection.

Consider that we have two ADSs with the same configuration. The camera image stream of ADS_1 and ADS_2 is denoted by $I_{1}$ and $I_{2}$, respectively, and both image streams are acquired by camera sensors over the same geographical location but from different angles and positions. Let $M_{k\;}\left(P_{I1,i},\;P_{I2,j}\right)$ be a matched keypoint between the keypoint $P_{I1,j\;}\left(x_{I1,j},\;y_{I1,j}\right)$ of image $I_{1}$ and the keypoints $P_{I2,i\;}\left(x_{I2,i},\;x_{I2,i}\right)$ of image $I_{2}$. This research aims to develop a collaborative perception based on image fusion and stitching techniques. Our proposed approach can be intuitively deployed to multiple ADSs. However, the computational and communicational costs may slightly increase.

#### 3.2.1. SIFT-Based Feature Extraction

In the SIFT algorithm, the first step is to identify a set of matched keypoints between the image streams $I_{1}$ and $I_{2}$ to be used for image fusion. Using the gamma correction, the contrast of the images $I_{1}\;$and $I_{2}$ are increased to detect the keypoints correctly. As the SIFT-based image fusion and stitching techniques require keypoints in the form of blobs, each keypoint $P_{I1,i}$ and $P_{I2,j}$ are then extracted in blob form from each image pair. The feature vectors of keypoints $P_{I1,i}$ and $P_{I2,j}$ are assigned, which are based on neighboring pixels that determine the descriptors $D_{I1,i}$ and $D_{I2,j}$, respectively. 

The SIFT algorithm uses the difference of Gaussian (DoG) scale-space to extract the features. In the scale-space function, the original image with the Gaussian function is convolved to construct the multiple levels of Gaussian pyramids; the scale-space keypoints are detected in the constructed Gaussian pyramids. Once the DoG of the image streams $I_{1}$ and $I_{2}$ are obtained, the keypoints are identified by comparing each pixel in the DoG images with its neighboring regions. Let us consider that we have an image stream Img from an ADS. During the feature extraction through SIFT algorithms, it first starts searching for stable feature points from the image stream Img across all potential scales in the scale space. Consider that we have an image frame Img(x,y) of ADS with the pixel coordinate (x,y). The scale-space for the input image Img(x,y) can be defined as follows:

(1) L(x,y,σ)=G(x,y,σ)×Img(x,y)

where L(x,y,σ) is the Gaussian transformation, σ is the scale-space factor, and Img is the input image stream from the ADS camera, while G(x,y,σ) is driven by Gaussian distribution, which is given as follows:

(2) $$G\left(x,y,\sigma \right)=\frac{1}{2{\pi \sigma }^{2}}e^{-\left(x^{2}+y^{2}\right)/(2\sigma^{2})}$$

To detect the position of keypoint features of the input image Img in the scale space, the establishment of the DoG pyramid is crucial in SIFT. The DoG can be established by taking the difference in the nearby scales as follows:

(3) D(x,y,σ)=(G(x,y,kσ)−G(x,y,σ))×Img(x,y)

From Equation (1),

L(x,y,σ)=G(x,y,σ)×Img(x,y)

Therefore,

(4) D(x,y,σ)=L(x,y,kσ)−L(x,y, σ)

where the constant *k* is the proportional factor between two adjacent scales. 

Each pixel of the image frame from the ADSs DoG images was compared with its neighbors at the current scale and adjacent scales to detect the key features invariant to the scale and orientation.

The candidate feature points extracted from the camera images of ADSs have to be refined to determine the location and the scale. This allows some feature points to be discarded that have low contrast and are poorly localized. Let us consider that $\hat{X}$ be the candidate feature point, which is selected to discard the feature points that have low contrast and are poorly localized in the ADSs camera images. The Taylor expansion of the DoG function for D(x,y,σ) produces Equation (5) which is used to refine feature points for best fit to location and scale.

(5) $$D\;\left(\hat{X}\right)=D+1/2\left(\partial D^{T}/\partial \;X\right)\hat{X}$$

where $\hat{X}$ denotes the offset from extremum. All those extrema with a value of $D\left(\hat{X}\right)$ less than the threshold are discarded and rejected for further image fusion.

#### 3.2.2. Feature Matching, Image Fusion, and Blending

In the feature extraction phase, the SIFT algorithm detects the key feature points and assigns the location, scales, and orientation. Now, it is important to describe those features in a highly distinctive way but invariant to possible illumination and viewpoints in the highly dynamic driving environment. The keypoint descriptor is a unique identifier for specific keypoints. The SIFT algorithm uses the gradient magnitude and directions of the keypoints as a keypoint descriptor. The image fusion and stitching combine the two image frames into a single image frame. In our domain, the ADSs camera sensors’ image frames are shared with other ADSs within close vicinity to detect the occluded objects. Image fusion and stitching consist of two steps: feature matching and image blending.

In the feature-matching process, the descriptors of the keypoints in the image frames from multiple ADSs are compared. The criteria for matching the features are that if the difference between the descriptors of two keypoints (i.e., from different ADSs camera image frames) is below the thresholds, they turn into keypoint pairs. These keypoint pairs with a negligible difference between their keypoint descriptor are taken as reference keypoints, which are then stitched into one frame in the image blending process. The multiple-image frames from different ADSs are blended into a single image frame in the image blending. The pixel values in the overlapping region are equal to the average weighted values of the blended frames. Consider that we have two ADSs image frames $I_{1}$ and $I_{2}$, respectively. The pixel values in the overlapping region can be obtained using Equation (6).

(6) $$P_{val}=\frac{D_{I1}}{D_{I1}+D_{I2}}P_{I1}+\frac{D_{I1}}{D_{I1}+D_{I2}}P_{I2}$$

where $D_{I1}$ and $D_{I2}$ are the distance of the overlapped pixels from the edge of the image $I_{1}$ of ADS_1 and image frame$\;I_{2}$ of the ADS_2, respectively. While $P_{I1}$ and $P_{I2}$ are the pixel values of the image frame $I_{1}$ and $I_{2}$ in the overlapping region. $P_{val}$ is the pixel value of the overlapped region after image frames are fused and stitched. 

As SIFT detects and generates much more abundant feature points, those features can sometimes be mismatched. These inaccurately matched features can affect the geometric transformation of the fused image. The RANSAC algorithm removes the mismatched feature points to improve the accuracy and quality of image frame stitching. The RANSAC algorithm estimates the model’s parameters from observed data containing outliers through an iterative approach and finds optimal fitting by removing outliers.

#### 3.2.3. Collaborative Perception and Object Detection

Object detection models have achieved tremendous accuracy and precision in recent years. Despite achieving high accuracy and precision, these models still have limitations in detecting occluded objects and objects beyond the sensors’ field of view. Various solutions have been proposed to overcome these limitations, such as homogenous sensor data fusion, heterogeneous sensor data fusion, and co-operative perception.

**Figure 3:**
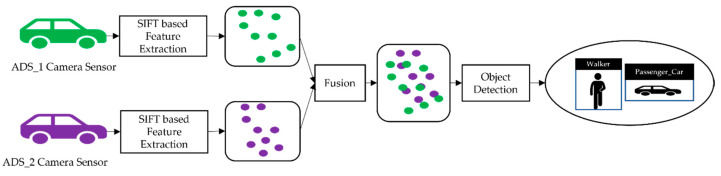
Collaborative perception systems and joint object detection based on Faster R-CNN.

The image fusion, stitching algorithm, and real-time object detection model considered in our approach require camera sensor data. Each camera sensor on ADSs generates the image frames, which are then processed through the SIFT algorithm to extract features for feature-based fusion. The image fusion allows the aggregation of information about the object in the detection zone through the collaboration of spatially diverse observation, which detects the occluded object and objects having low visibility to the sensor. The image fusion phase begins with processing each sensor’s image on the local processor of ADSs. It first extracts the features from the image and then compresses it to transmit to the nearby by ADSs in VCPSs for collaborative perception. When each image frame is transmitted to other ADSs, it is concatenated with the image frames of receiving ADSs into one image frame. The concatenated image frames are then fed to the object detection model based on Faster R-CNN to detect, classify, and localize the object in the scene. The collaborative object detection system returns a list of the detected objects with bounding boxes. Figure 3 shows our proposed collaborative perception system’s fusion scheme and object detection system.

The joint object detection system in our collaborative perception system is based on Faster R-CNN. Faster R-CNN follows the multi-task learning procedure that combines classification and bounding box regression for object detection. It uses a convolutional backbone, such as visual geometry group (VGG), and residual neural network (ResNet) in the feature extraction process from the input images. The Faster R-CNN consists of two stages: (1) a region proposal network and a (2) Fast R-CNN header network. The RPN uses the extracted features to predict the class-agnostic box proposal, such as objects or backgrounds. The class-agnostic box proposal is achieved by predicting multiple candidate boxes for each location by using multi-scale reference anchors. However, fewer proposals are selected as the region of interest (ROI) and forwarded to the header network. These selected ROIs are used as a base for cropping the features through an ROI pooling operation. The cropped feature maps are then fed to the backbone network to predict a class and the bounding boxes around the detected object. As Faster R-CNN shares the convolutional features between both stages, the accuracy and detection speed is comparatively faster than the existing approach. After getting the feature fusion, these features are passed to the RPN, which produces two outputs. The first output is the loss function that calculates the classification and regression losses. The second output is the probability score of the proposed region of interest and the location of the proposed region. The probability score Pscore ∈[0,1] has a value between 0 and 1.

The location of the proposed regions can be defined as $P=\left(P_{x},{\;P}_{y},{\;P}_{z},{\;P}_{\theta },{\;P}_{l},{\;P}_{w},{\;P}_{h}\right)$, where $P=\left(P_{x},{\;P}_{y},{\;P}_{z}\right)$ is the center of the proposed region and $\left(P_{\theta },{\;P}_{l},{\;P}_{w},{\;P}_{h}\right)$ defines the rotation angle, length, width, and height.

## 4. Experimental Setup

We used the CARLA simulator to evaluate the proposed collaborative perception system. It allows us to simulate complex driving environments. Additionally, it also helps in obtaining ground truth data to train and evaluate deep learning models. The dataset we collected from the CARLA simulator for collaborative perception can be used to train and evaluate the real-time object detection and localization models. For this experiment, we simulated the collaborative perception using two ADSs. These two ADSs share their sensor information to increase their situational awareness by detecting more occluded objects. In the first phase, the camera sensor data are collected from different simulation environments, and the Faster R-CNN model is trained for real-time object detection and localization. Once the model is trained, the models are deployed on each ADSs. The feature extraction of the camera sensor image frames of each ADSs, along with shared image frames, is done using the SIFT feature extractor model. The extracted features are then fused to detect and localize the object using Faster R-CNN. The proposed system was evaluated in different traffic scenarios such as roundabouts, T-junctions, intersections, and multilane roads. We used the Ubuntu16.04 64-bit system with NVIDIA GeForce RTX2060 GPU for our experiment. The system was equipped with a core i7 processor and 32 GB RAM for the experimental setup. In the following, we describe the experimental setup in detail.

### 4.1. Dataset

The dataset used in our experiment was obtained using the CARLA simulator. It contains camera images of mixed traffic environments such as roundabouts, T-junctions, intersections, and multilane roads. We chose these testing scenarios because such driving scenarios are challenging for ADSs, and complex driving maneuvers are needed to drive in such complex scenarios. Occluded objects, pedestrians, and cars in these driving scenarios pose serious safety concerns for the ADSs and manual-driving cars. In contrast to the existing approaches for collaborative perception, where the sensors are fixed on roadsides [33], we used the camera sensors mounted on ADSs to generate the dataset for our experiments. 

We configured the camera sensors of two ADSs in CARLA simulators to gather the desired data. As the ADSs can move independently of each other, we can test entire driving scenarios in simulated environments. Overall, we collected 1895 image frames of different driving scenarios, and we prepared our own 1895 labeled simulation-based images that can be used for object detection in real-time in the CARLA simulator. This can also be used to evaluate the performance of the real-time object detection algorithm in collaborative perception. The dataset contains four classes: *walker*, *passenger*_*car*, *truck*, *motorcycle*, and *cycle*. We split the dataset into the training set, consisting of 1713 labeled images and 182 images for the test set. The data augmentation, including rotating, flipping, zooming in, and zooming out, was done on the original dataset to overcome the problem of imbalanced data. (The dataset can be made available upon request.)

### 4.2. Testing Scenarios

Using the dataset we collected from the CARLA simulator, we simulated a list of different driving scenarios such as intersections and roundabouts, T-junctions, and multilane roads. Road intersections and T-junctions are among the most challenging driving scenarios where vehicles are congregated and thus cause occlusion. In such scenarios, the camera sensor-based ADSs face serious safety concerns as the camera’s vision are blocked due to the occlusion caused by the vehicle in front, and the situational awareness of the ADSs becomes severely limited. We used these driving scenarios to validate our proposed collaborative perception. 

Another testing scenario to validate the proposed collaborative perception systems is multilane roads and roundabouts. Such roads are always prone to accidents due to the combination of high-speed driving. Early detection of moving cars and other objects through perception sharing may increase road safety. Therefore, we considered the roundabout driving and multilane road as a testing scenario to validate the performance of the proposed system.

### 4.3. Training Process

We trained the object detection model for each scenario using the fused camera image features of multiple ADSs. This paper used the Faster R-CNN inception-v2 pre-trained model on the dataset collected, as described in Section 4.1. To accelerate the training process and reduce the overfitting, the weights of each batch normalization layer in the pre-trained model (i.e., Faster R-CNN inception-v2) are kept frozen. First, the RPN is trained using the fused images on a minibatch, and the RPN and base network parameters are updated. Once the base network and RPN parameters are updated, then the positive and negative proposals generated by the RPN are used to train and update the classifier. The Faster R-CNN inception-v2-based classifier and RPN share the base convolutional layers. We used the same method to calculate the loss function and parameterization method of bounding box regression as those in the original Faster R-CNN model. The Adam optimizer [40] is used to optimize the loss function. During the training, the learning rate was set to 10^−3^. The network was trained for the 30,000 global steps.

## 5. Performance Evaluations

### 5.1. Research Questions

We considered the following research questions to evaluate the proposed collaborative perception system.

- *RQ1 (Effectiveness):* How effective is the proposed collaborative perception system in detecting occluded objects?
- *RQ2 (Performance):* How does the collaborative perception system precisely detect occluded objects and affect the model’s detection performance?
- *RQ3 (Safety Analysis):* What is the impact of collaborative perception on the safety of ADSs? How does the logistic chaos map-based encryption enhance safety in the collaborative perception system?
- *RQ4 (Comparative Analysis):* How does the proposed collaborative perception system compare with the detection confidence of Cooper [32] and F-Cooper [10]?

We evaluated the performance of the collaborative perception systems for object detection based on Faster R-CNN through a series of experiments. The testing scenarios used to evaluate the performance of the proposed systems are T-junctions, roundabouts, intersections, and multilane roads. The evaluation of the proposed systems was carried out on the collected dataset and the evaluation metrics such as intersection over union (IoU), mean average precision (*mAP*), and recall are used.

### 5.2. Evaluation Metrics

We used the IoU, precision, recall, and *mAP* evaluation metrics related to object detection to evaluate the proposed system. Additionally, the average data volume exchange between the ADSs for collaborative perception was measured in kilobit (kbit). The IoU measures the spatial similarity between the estimated ground truth boxes and the actual ground truth set. The IoU can be defined as follows:

(7) $$IOU\;\left(Bb_{AGT},\;Bb_{EGT}\right)=\frac{area\left(Bb_{AGT}\cap Bb_{EGT}\right)}{area\left(Bb_{AGT}\cup Bb_{EGT}\right)}$$

where $Bb_{AGT}$ and $Bb_{EGT}$ are defined as the actual ground truth and the estimated ground truth bounding boxes. $Bb_{EGT}$ includes a set of all positive boxes identified by the Faster R-CNN-based object detection model. Each bounding box has confidence above the set thresholds. The IoU takes the size, location, and orientation of both bounding boxes (i.e., $Bb_{AGT},\;Bb_{EGT}$). The value of IoU ranges between 0 and 1, where IoU is defined to be 0 if the $Bb_{AGT},\;Bb_{EGT}$ both do not have any overlapping regions. On the other hand, if the value of the IoU is 1, then it means the location, size, and orientation of both $Bb_{AGT},\;Bb_{EGT}$ are equal and completely overlapped. When the value of the IoU metric for the $Bb_{AGT},\;Bb_{EGT}$ is above a certain threshold, then$\;Bb_{EGT}$ can be defined as the matching estimation of $Bb_{AGT}$. Note that we can set the IoU threshold by our choice. A typical value for the threshold can be 0.5, 0.7, or 0.95.

The precision metrics used in our systems are average precision (*AP)* and *mAP*. The precision metric is the ratio of matched estimated bounding boxes to the total number of bounding boxes in the estimated set by the model. Similarly, the recall metric can be obtained by taking the ratio of matched estimated bounding boxes to the total number of bounding boxes in the ground truth set. The *AP* is the weighted sum of all precision at each threshold which can be defined using Equation (8). The weight is defined as an increase in recall.

(8) $$AP=\overset{k=m-1}{\underset{k=0}{\sum }}\left[recall\left(k+1\right)-recall\left(k\right)\right]\times precision\left(k\right)$$

where the m is the total number of the estimated boxes and the recall value $recall_{i}$∈ ($recall_{1},\ldots \ldots ,\;recall_{k}$) in Equation (8) can be obtained by assuming that the thresholds and the confidence score of the bounding box at $k^{th}$ position are equal.

The mAP can be obtained by using the AP for each class considered in the object detection model. The mean of all classes is the mAP which can be obtained by Equation (9).

(9) $$mAP=\frac{1}{m}\overset{k=m}{\underset{k=0}{\sum }}AP_{k}$$

where $AP_{k}$ is the average precision of the class k, and m is the total number of classes. 

### 5.3. Top-Level Performance Evaluation of Proposed System

**Figure 4:**
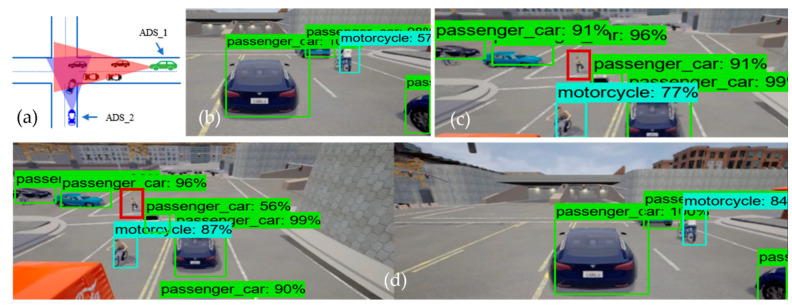
Top-level performance analysis of individual perception system of ADS_1 (receiver), ADS_2 (sender), and collaborative perception of ADS_1 at an intersection scenario. (**a**) represents an intersection scenario driving scenario, (**b**) represents the perception of ADS_1, (**c**) represents the perception of ADS_2, and (**d**) represents the collaborative perception of ADS_1.

**Figure 5:**
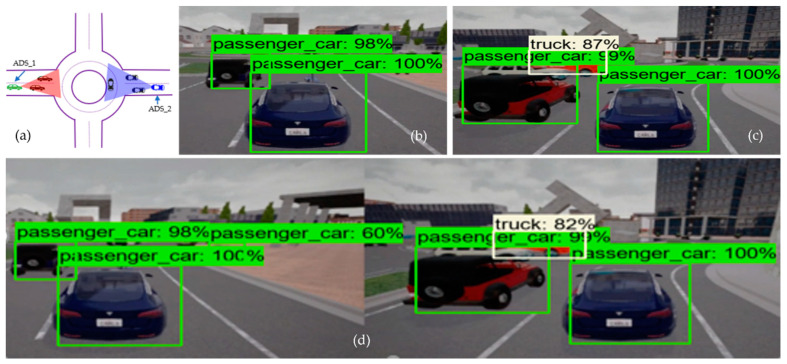
Top-level performance analysis of individual perception system of ADS_1 (receiver), ADS_2 (sender), and collaborative perception of ADS_1 at a roundabout driving scenario. (**a**) represents a roundabout driving scenario, (**b**) represents the perception of ADS_1, (**c**) represents the perception of ADS_2, and (**d**) represents the collaborative perception of ADS_1.

In order to evaluate the performance of the collaborative perception, we analyzed the performance of the models individually as well as the performance of the model in collaborative perception. We used the term “top-level performance evaluation” as an alternative to the visual analysis of proposed systems. We can see the result of the image fusion of two ADSs in Figure 4 and Figure 5, with receiving ADS (i.e., ADS_1) and data sender ADS (i.e., ADS_2). Figure 4 illustrates the performance of the object detection model at the intersection testing scenario, where Figure 4a is the bird-eye view of the road intersection. Figure 4b shows the detection result of ADS_1 (i.e., the receiver), just as Figure 4c represents the sender ADSs (i.e., ADS_2) perception. Finally, Figure 4d shows the detection result of the collaborative perception system of ADS_1. The performance evaluation was done based on the confidence threshold of 0.5, 0.7, and 0.95 for the object detection model on the individual system as well as the object detection model used in collaborative perception. Figure 4 and Figure 5 illustrate the top-level performance of the proposed systems with a confidence threshold of 0.7. When the confidence is above 0.7, the object detection model marks the bounding box for the detected object. In Figure 4d and Figure 5d, we can see the detection results of the object detection model used in collaborative perception for intersection and roundabout testing scenarios.

From Figure 4b, we can see that the ADS_1 could only detect four objects (i.e., one motorcycle and three passenger cars), and Figure 4c shows that the ADS_2 approaching the intersection from another road could detect five objects (i.e., one motorcycle and four passenger cars). However, it would be beneficial for both vehicles to know the information regarding the static and dynamic objects on their future trajectory, and such information may increase the situational awareness of both cars in terms of planning and decision. This can be achieved by fusing the perception of each ADS into collaborative perception where both the camera sensor data are fused in order to detect more objects, including occluded ones. Figure 4d clearly illustrates the effectiveness of the collaborative perception system where the receiver ADS can detect more objects on the road that its sensors could not detect. Taking a closer look, we can see that there is occlusion for both ADSs. Those objects in the field of view of ADS_1 are not visible to ADS_2 and vice versa. Hence, by sharing the perception of ADS_2 with ADS_1, the ADS_1 can see the object on its future trajectory, which helps the ADS_1 make better planning and decisions. However, this is not the same for all cases, as some objects could not be detected by either ADS_1 and ADS_2 or the collaborative perception. The red-colored bounding box illustrates that neither ADSs detected the pedestrian and their collaborative perception. There could be many reasons for not detecting these objects, for instance that the object detection models’ performance varies with the object’s size. Other reasons could be the occlusion caused by other comparatively big objects.

Similarly, in the roundabout driving scenario, where two ADSs are approaching the roundabout from the opposite direction, it would be suitable for both ADSs if they had more information about their surroundings. From Figure 5b, we can see that ADS_1 detects only two passenger cars, and ADS_2 (i.e., Figure 5c) detects three objects (two passenger cars and one truck). However, after the fusion of the camera sensor data of both ADSs, we can see that the detection rate of ADS_1 increased drastically. Figure 5d presents the collaborative perception of the receiver car (i.e., ADS_1) in the roundabout driving scenario. By closely inspecting the collaborative perception of ADS_1 at the roundabout scenario, we can see that it detected six objects, including one passenger car, that both ADS_1 and ADS_2 did not detect by their individual perception systems. From these comparisons, we can conclude that the average detection rate of collaborative perception was 45.4% more than the individual perception system of ADSs. The fusion of multiple ADSs camera sensor data increased the detection rate of occluded objects compared to individual perception systems.

### 5.4. Quantitative Performance Analysis of Collaborative Perception System

After taking the overview of the top-level performance analysis of our proposed collaborative perception systems, we now dive into the quantitative analysis of collaborative perception compared to the individual perception systems.

The data used to evaluate the collaborative perception comes from the dataset we collected using the CALRA simulator for testing scenarios. The results were reported using the IoU thresholds at 0.5, 0.7, and 0.95 for object detection. When the object detection confidence score for any object is above the IoU threshold, the model marks the bounding box on that object. The precision was calculated by comparing the detected objects with their ground truths using the evaluation metrics mentioned in Section 5.2. The quantitative performance analysis was carried out for intersections, T-junctions, roundabouts, and multilane road-testing scenarios.

**Figure 6:**
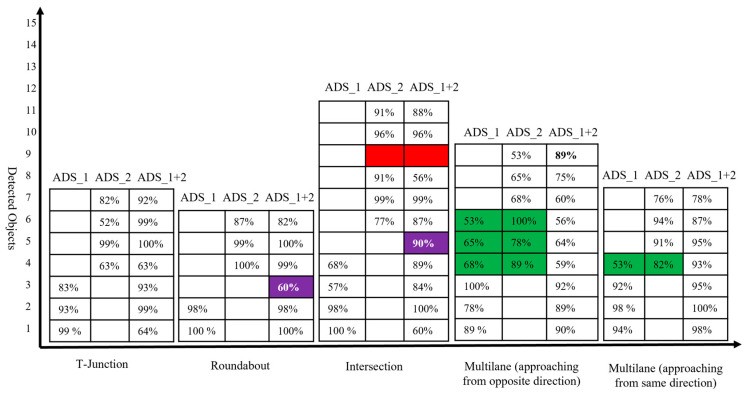
The intuitive detection result of ADS_1, ADS_2, and collaborative perception of ADS_1. The table presents the detection confidence in the percentage of each ADSs, as well as the collaborative perception of ADS_1.

*Effectiveness (RQ1):* To answer the research question RQ1, we present the experimental results in Figure 6 and Table 1. Table 1 and Figure 6 report the efficacy of collaborative perception in detecting occluded objects on *mAP* metrics. It also reports the superiority of collaborative perception over individual perception systems. The results show that the detection performance increased in terms of *mAP*. We observed that the precision of the collaborative perception system was increased when the IoU threshold was set to 0.5. For example, in the T-junction driving scenarios, the increase in *mAP* was 6.3% compared to ADS_1, and 5.2% compared to ADS_2. As in the intersection scenario, the gain in *mAP* was 5.9% compared to ADS_1, and 4.6% compared to ADS_2. The same trends were recorded in another driving scenario (i.e., *multilane road (same direction)*). The increase in *mAP* was 7% compared to ADS_1, and a 6.4% gain in *mAP* was recorded compared to ADS_2. However, it can be observed that there are some variations in detection performances. For example, when IoU = 0.7 at T-junction, the gain in *mAP* of collaborative perception was only 0.1% compared to ADS_1.

In most cases, no significant increase was observed in *mAP* when the IoU threshold was set to 0.7. Only in two scenarios (i.e., *roundabout and multilane road (same direction)*) the *mAP* was increased up to 6.7% compared to ADS_1, and a 6.2% increase in *mAP* was observed compared to that of ADS_2 in the roundabout scenario. As in the *multilane road (same direction)* testing scenario, the gain in *mAP* was observed at 5% compared to ADS_2, while a 5.9% increase in *mAP* was recorded compared to ADS_1. When we set the IoU threshold to 0.95, we observed a significant gain in all five driving scenarios except in *multilane road (opposite direction)* driving scenarios. The gain in *mAP* does not increase further as the performance gain reaches its saturation levels. The performance gain may increase if participating ADSs in collaborative perception systems increases. However, the average object detection rate of collaborative perception is 45.4% more than the individual perception system of ADS_1 and ADS_2, as shown in Figure 6. We use only the front camera images for fusion. However, the front camera of both ADSs does not cover the large detection areas. As the detection area increases, the participation of more ADSs in collaborative perception systems would need to maintain the increasing trends in the performance gain. Although the participating ADSs in collaborative perception systems were only two in our current experiment, we can still observe a significant increase in the precision and object detection rate. More participating ADSs in collaborative perception systems can increase detection precision; however, the computational cost may also increase. 

The location and alignment information of features for fusion has a significant impact on the object detection model’s performance. The change in alignment or translation may cause a bad impact on detection accuracy, despite the fact that in the dynamic driving environment, the precision of the object detection model is quite high in our experimental results. We can observe that the performance of the model in terms of *mAP* is stable. Meaning that there is no significant change in *mAP* with respect to driving scenarios on the same IoU thresholds. For example, the average difference between *mAP* at the same IoU thresholds is less than 4% in most cases. This shows the stability of object detection models in highly dynamic driving scenarios.

From Table 1, we can see that the detection confidence sometimes varies in different scenarios. It is the location and dynamic driving environment that causes the small variation in detection confidence. However, from Table 1, we can see that the object detection model performed extremely well in detecting objects in individual perception systems and collaborative perceptions. The highest *mAP* (i.e., 80.1% and 80.4% when the IoU threshold was 0.5) was reported on the individual perception system of ADS_1 and ADS_2, respectively. On the other hand, the highest *mAP* was recorded at 86.8% when the IoU threshold was set to 0.5 on the collaborative perception system of ADS_1. This indicates that the fusion of camera images increases the precision of object detection models. We believe that if the number of participating ADSs in collaborative perception systems increases, the *mAP* will also increase. 

*Performance (RQ2):* Moving to the comparative analysis of individual perception systems and collaborative perceptions in terms of detection rate, we can answer research question RQ2. The average number of the detected object in the collaborative perception system in each testing scenario is 45.4% more than in the individual perception system of both ADSs. Figure 6 depicts the number of objects detected by the ADS_1 and ADS_2 and the collaborative perception system of ADS_1. In Figure 6, the table presents the detection confidence in percentage in each testing scenario. The vertical axis represents the number of objects detected by each ADSs, and the collaborative perception of ADS_1 and the testing scenarios are given on the horizontal axis. The empty cell on the table represents that there are no more objects in the range of camera sensors. The red-colored cell represents the objects in the range of the camera sensor. However, the object detection models of both individual and collaborative perception systems could not detect them. The green-colored cells illustrate objects detected by both ADSs (i.e., all those objects in the range of both ADSs’ camera sensors.). Another important cell representation is the blue-colored cell. These blue-colored cells represent the objects that were not detected by the individual perception of both ADSs; however, due to the image fusion of both ADSs, the collaborative perception system detected those objects. 

From Figure 6, we can see that the collaborative perception systems outperformed the individual perception systems of both ADSs. Starting from the T-junctions testing scenario, it can be noted that the number of detected objects in the collaborative perception system of ADS_1 is two times more than its own perception system, as ADS_1 could only detect three objects by its own perception systems. Due to limited sensing range and occlusion, it could not detect more objects on its future trajectory. However, after fusing the camera sensor image of ADS_2, we can see a significant increase in the number of the detected object in collaborative perception. Now, the ADS_1 can detect four more objects those were not in its sensor range. As in the roundabout testing scenario, the collaborative perception system of ADS_1 detected four more objects due to image fusion. An interesting phenomenon was observed in both roundabout and intersection testing scenarios. In each case, the collaborative perception system detected one extra object, which was not detected by the individual perception systems of both ADSs. This phenomenon was very unexpected and interesting to analyze in our experiment, and the detected object and their confidence are given blue-colored cells in Figure 6.

The intersection testing scenario has another interesting phenomenon to analyze, as expected. The collaborative perception system detected more objects (i.e., 10 objects compared to ADS_1, which detected only four objects, and ADS_2, which detected only five objects). However, one extra object (i.e., the blue-colored cell in Figure 6 in the intersection testing scenario) that has 90% detection confidence has been detected. Another unique phenomenon to analyze in the intersection testing scenario is the red-colored cell representing objects that were not detected by either individual perception systems of ADSs or collaborative perception. Upon careful investigation of simulation logs and results, we observe that there was a pedestrian (i.e., the red bounding box in Figure 4c,d) in the range of the camera sensor of ADS_2. However, it could be detected by neither the object detection model of ADS_2 nor the object detection model of the collaborative perception. There may be plenty of reasons for not detecting such objects. Firstly, this may happen due to the occlusion due to other big objects. As we can see in Figure 4c,d, the pedestrian is surrounded by other big objects compared to its size and therefore causes occlusion and results in the pedestrian not being detected. Another reason is that the object detection models’ performance varies with the objects’ size. The object detection model performs worse when detecting small-sized objects than large objects [11]. As expected, the collaborative perception system also outperformed the individual perception system of ADS_1 and ADS_2 in detecting more objects in *multilane (approaching from the opposite direction)* and *multilane (approaching from the same direction)* road testing scenarios. As we can see, some objects (i.e., cells with green color) in Figure 6 were detected by both ADS_1 and ADS_2. We believe that these phenomena are due to the specific driving scenarios, as these objects were in the range of camera sensors of both ADS_1 and ADS_2. However, only a single instance of these redundant objects is detected in a collaborative perception system. Hence, this proves the effectiveness of the proposed collaborative perception, meaning that if more than one ADSs driving system detects a single object, then only a single instance of that object is detected in the collaborative perception.

### 5.5. Analysis of Communication Cost and Computational Time

Having taken the overview of top-level performance analysis and the quantitative analysis of proposed collaborative perception systems, we now dive into a detailed analysis of communication costs metrics (i.e., kbit) and computation time (i.e., ms) for all testing scenarios. As the fusion scheme for all testing scenarios was the same, therefore, there was no significant change observed in communication cost. After compression, the average image frame size was 357 kbit in all testing scenarios, which is quite feasible for low computational powered systems. The average frame rate was set to 10 frames/s during the simulation. The required communication link capacity to send the camera sensor image frame from one ADS to another for fusions depends on the processing frame rate. We set the frame rate in our simulations at 10 frames/s. This means that the image fusion of two ADS for a collaborative perception system with a processing rate of 10 frames/s would require a communication link with a capacity of 3.57 Mb/s (i.e., 357 kb/frame times 10 frames/s). We did not consider the communication delay as the detailed investigation of communication delay is beyond the scope of this article. 

However, the transmission rate of 10 frames/second can be easily supported by commercially available wireless communication links. Table 2 presents the communication cost and computational time required to process the image fusion of two ADSs. As we can observe, each frame with a size of 357 kbit requires only 301 ms for the whole process (i.e., from the transmission of image frames to inference). However, the computational time is dependent upon the hardware specification. In our experiment, we used NVIDIA GeForce RTX2060 GPU with 6GB GPU dedicated memory, and the system was equipped with core i7 processor and 32 GB RAM. During the experiment, we observed the total GPU utilization was 75%.

### 5.6. Impact of Collaborative Perception on Safety

To answer RQ3, we analyze the impact of collaborative perception on safety. While performing the safety analysis of collaborative perception, we consider a safety-critical scenario presented in our motivation, i.e., Figure 1b. The safety analysis in this article is based on time-based risk assessment methods. Generally, the forward collision algorithms with risk assessment methods have two approaches: time based and distance based. However, we considered a time-based approach in our experiment as time-based risk assessment methods are proven effective on the road [41]. Time-based risk assessment is based on time-to-collision (TTC). In our collaborative perception, we used a forward collision avoidance algorithm based on a time-based risk assessment method to avoid collisions with pedestrians by considering the scenario presented in Figure 1b. We simulated the scenario using two cars (i.e., ADS_1, the sender, and ADS_2, the receiver). To analyze the impact of collaborative perception on safety in such scenarios, we present two scenarios: hazardous scenarios and safe scenarios. In the following, we explain each scenario.

**Figure 7:**
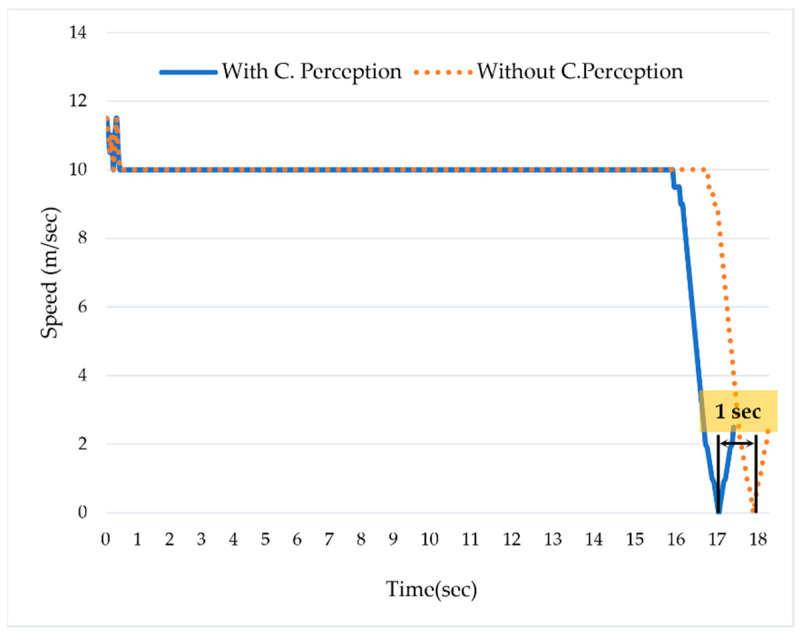
The behavior of an ADS with and without collaborative perception.

In order to simulate the hazardous scenario, we did not use the collaborative perception system. The ADS_2 was moving forward with a speed of 10 m/s. Due to occlusion caused by the bus, the ADS_2 could not detect the pedestrian, and, as a result, caused a hazardous scenario. Despite applying the emergency brake, we confirmed that the distance between the ADS_2 and the pedestrian was close to zero. This hazardous scenario was analyzed using the velocity logs and the distance between the ADS_2 and the pedestrian. Figure 7 shows that the ADS_2 was moving with a speed of 10 m/s; the ADS_1 detected a pedestrian in the middle of the road at 16th seconds. However, as the ADS_2 was not using the collaborative perception, it responded to the situation on the 17th. Despite applying the brake, the ADS_2 could not stop and collided with the pedestrian. We can see that the ADS_2 started deceleration at the 17th s and stopped at the 18th. Due to the late response, the safety distance between the ADS_2 and the pedestrian was close to zero.

In contrast to the hazardous scenario, we simulated the safe scenario using two ADSs that use collaborative perception. Figure 7 shows that the ADS_2 was moving forward with a speed of 10 m/second. The ADS_1 detected the pedestrian at the 16th s and shared its real-time perception with ADS_2 via collaborative perception. The ADS_2 proactively responded to the situation one second earlier compared to the scenario in which it was not using the collaborative perception. Additionally, after analyzing the safety distance (i.e., distance to collision) between ADS_2 and the pedestrian, we confirmed that the safety distance was 1.2 m. Figure 7 shows that ADS_2 proactively started reducing the speed at the 16th s right after detecting the pedestrian in collaborative perception. Therefore, it could stop at a safe distance, i.e., 1.2 m from the pedestrian. In contrast, while the ADS_2 was not using the collaborative perception, the safety distance was almost equal to zero. 

However, the collaborative perception system is always prone to attack from malicious vehicles sending phantom information, which may cause hazards for participating ADSs. Therefore, in order to avoid the phantom information sent by malicious vehicles in collaborative perception, we use the chaotic map-based encryption technique. The logistic map is a chaos system with highly complex behavior [42] and is very sensitive to the initial condition. Mathematically it can be described as:

(10) $$\begin{array}{c} x_{n+1}=r\times x_{n}\left(1-x_{n}\right)\\ f\left(x_{n}\right)=x_{n+1} \end{array}$$

The parameter r in the logistic map ranges between 0 and 4. $x_{0}\in \left(0,1\right)$ represents the initial value. Each iteration of the map in logistic chaos generates a value known as iterates. n=1,2,3…., N represents the number of iterates and the variable $x_{n}$ represents the chaotic output, and its values range between [0, 1]. The chaotic behavior in the logistic map can be achieved after several iterations. The value of r must remain less than the range to achieve chaotic behavior—the higher values of *r* cause impossible to achieve chaotic behavior [43].

In our proposed encryption scheme, we first generate the chaotic sequences followed by the confusion process in which the pixel values are confused and then shuffle the pixel position to generate the encrypted image. Consider that we have an image frame I of camera sensors of ADS_2, which is to be transmitted to ADS_1 via collaborative perception. M×N represents the size of the image frame, and the pixel of each image frame I is I(i,j). The I(i,j) represents the pixel value at the position (i,j). We obtained the initial value for the logistic map from the secret key. The secret key used in the chaotic map-based encryption consists of 256bits in ASCII form. After defining the initial condition, we transformed the I(M×N) into an array and converted each pixel value to an integer ranging from 0–255. Following by transformation process, we generated the chaotic sequence $x_{i}=\left(x_{1},x_{2},\ldots .,\;x_{n}\right)$ using the equation $x_{n+1}=r*x_{n}\left(1-x_{n}\right)$. The confusion was achieved via XOR operation using the equation $C_{i}=P_{i}⊕x_{i}$, where the variable $P_{i}$ denotes the array of pixel values. Finally, we shuffle the pixel values to get the encrypted image. For the decryption process, we follow the inverse of the encryption process as the decryption is the inverse of the encryption process.

The logistic chaos map-based encryption and decryption process can be summarized as follows: first, the pixel values of each image frame of the ADS_2 are transformed into an array, and secondly, we convert the pixels’ values into an integer between a range of 0 to 255 using mod operation. The third step is to generate the chaotic sequences, and finally, in the fourth step, confusion and diffusion are performed to get the required encrypted image. In contrast, the inverse encryption process is followed at the receiving end (i.e., ADS_1) to get the decrypted image.

**Figure 8:**
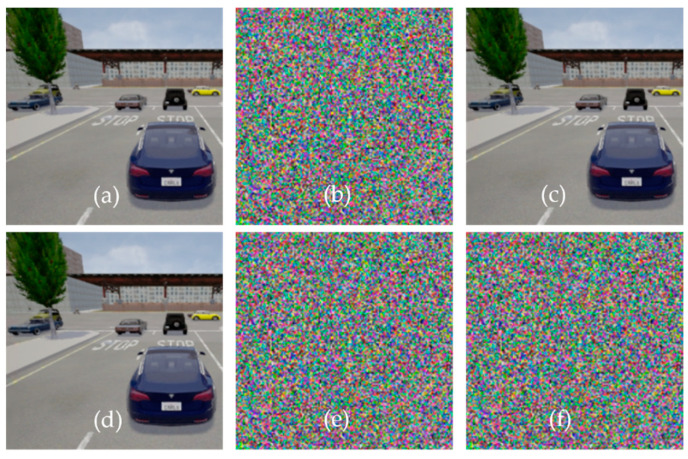
Logistic map-based encryption system. (**a**–**c**) represent the original, encrypted, and decrypted image frames with the same key, respectively, while (**d**) represents the original, (**e**) the encrypted, and (**f**) the decrypted image using the incorrect key.

In response to the second part of the RQ3, we analyze the impact of chaotic map-based encryption on safety by considering the key sensitivity, histogram, and adjacent pixel autocorrelation analysis. During the experiment, we set the initial value for the chaotic map at 0.1, and the value of the variable *r* was set to 3.76. We used the 256-bit, i.e., 32 characters long encryption key. The image frames of ADS_2 were encrypted using the encryption key 1q2w3e4r5t6y7u8i9o0p!@#$%^&*(){} and these image frames were shared with ADS_1. At receiving end, each image frame was decrypted using the same key. Figure 8a–c depicts the encryption process and decryption process using the same key. We can see that there is no visual difference between encrypted and decrypted images when the key is the same for encryption and decryption.

The encryption and decryption technique should be key sensitive, and the system must produce entirely different patterns for any change in the key. From Figure 8d–f, we can see that the proposed encryption technique is highly key sensitive. A minor change in the key produced an entirely different pattern and produced no visible information in the decrypted image. The encryption key was set to 1q2w3e4r5t6y7u8i9o0p!@#$%^&*(){}, and while decrypting the image, the key was set to !q2w3e4r5t6y7u8i9o0p!@#$%^&*(){}. Despite the fact that the difference between the encryption (1q2w3e4r5t6y7u8i9o0p!@#$%^&*(){}) and decryption key(!q2w3e4r5t6y7u8i9o0p!@#$%^&*(){}) was only one character (i.e.,!), the decrypted image contains no visual information. This indicates that the encryption scheme is very key-sensitive. If any malicious vehicle tries to access or send phantom information, the encryption technique will avoid such phantom information, thus enhancing safety.

**Figure 9:**
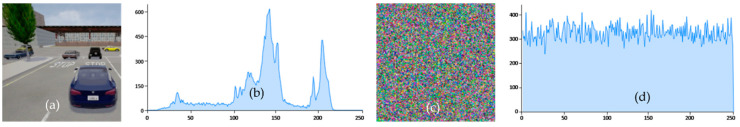
Histograms of original and encrypted image frames. (**a**) represents the original sample image frame, (**b**) depicts the histogram of the original image while (**c**) represents the encrypted image frame, and (**d**) is the depiction of the histogram of the encrypted image frame.

The histogram analysis of both encrypted and decrypted image frames proves that our logistic map-based encryption techniques provide enough safety and security from malicious vehicles. Histograms describe the distribution of image pixels, and a good encryption technique must produce a uniform histogram for all encrypted image frames. Figure 9 shows the histogram of the original (i.e., Figure 9b) and encrypted image (i.e., Figure 9d). As it can be seen that the histogram of the encrypted image has more uniform spikes as compared to the original image. We can see that the histogram of the original image is not uniform. These figures show no statistical similarity between the original and encrypted images, consequently providing no meaningful information for the malicious vehicle. 

**Figure 10:**
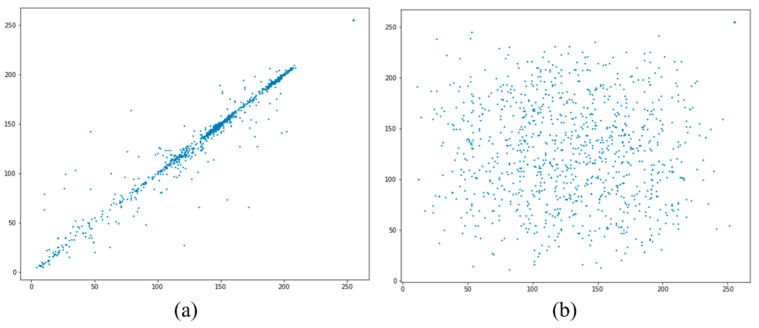
Adjacent pixel correlation graph of original and encrypted image frames. (**a**) adjacent correlation graph of the original image and (**b**) adjacent correlation graph of the encrypted image.

To avoid the attack from malicious vehicles on collaborative perception, the adjacent pixels in the encrypted image should be noncorrelated and the value of correlation should be close to zero. Higher correlation implies higher similarity between adjacent pixels, and Figure 10 shows that the original image has a higher correlation than the encrypted image. From Figure 10a, the correlation between the adjacent pixel is high in horizontal, vertical, and diagonal positions. In contrast, Figure 10b shows a correlation graph of the encrypted image, indicating that the correlation between adjacent pixels is very low. The correlation graphs of original and encrypted image frames confirmed that the encrypted image achieved zero correlation, proving that the encryption technique is robust against correlation attacks of malicious vehicles.

### 5.7. Comparative Analysis with Existing Works

*Comparison (RQ4):* To compare our approach with the existing fusion-based collaborative/co-operative perception system, we choose F-Cooper [10] and Cooper [32]. However, the direct comparison of our approach with these fusion methods would not be meaningful due to the sensor type, position, and data used for fusion. Table 3 presents the high-level comparative analysis of our approach with the existing baseline approaches. Additionally, we compare the object detection performance of Cooper and F-Cooper with our approach. F-Cooper reported their detection result in average precision. Both F-Cooper and Cooper reported the detection precision in two categories based on the distance between the camera sensor and the detected objects. The first category is the “Near” category, representing the object near the camera sensors. The second category is the “Far” category, representing the object far from the camera sensors. The cutoff between the “Near” and “Far” is 20 m. Regarding the detection performance of both F-Cooper and Cooper in multilane road-testing scenarios, the voxel feature fusion method was reported as the best-performing method from F-Cooper, with an average precision of 77.46% and 58.27% for the “Near” and “Far” category, respectively, when the IoU threshold was set to 0.7.

In contrast to the Cooper and F-Cooper, the detection confidence of our collaborative perception system was reported in *mAP*. Compared to the average detection precision of F-Cooper, our collaborative perception system *mAP* was reported at 79.9% in the multilane road-testing scenario. As in the road intersection scenario, the average detection precision of F-Cooper and Cooper reported for the voxel fusion method was 80.21% for the “Near” category and 72.37% for the “Far” category, respectively. On the other hand, the *mAP* for the road intersection scenario in our collaborative perception system was reported at 78.8%. Although we reported the detection confidence in mean average precision, while F-Cooper and Cooper still reported detection confidence in average precision, our detection confidence outperformed both F-Cooper and Cooper in multilane road-testing scenarios when the IoU threshold was set to 0.7. We also evaluated the performance of collaborative perception with the IoU threshold at 0.5 and 0.95. The result showed that the detection confidence when the IoU threshold was set to 0.5, our proposed approach outperformed F-Cooper and Cooper in both testing scenarios. For example, the *mAP* was 84.2% of our approach compared to F-Cooper, and Cooper’s average precision was 80.21% for “Near” and 72.37% for “Far” in the intersection scenario. Compared to the existing approach, each frame size in our proposed system was reported as less than 358 kbit, and the time required to process the image frames required less than 301 ms. We also compared the sensor placement techniques with [33], in which all the sensors in co-operative perception were placed in fixed positions (i.e., roundabouts and T-junctions). The objects beyond the range of these fixed positioned sensors are still unknown to ADSs, posing safety threats. In contrast, the sensor placement in our approach is not location dependent, meaning that the camera sensors are mounted on the ADSs themselves, enabling them to extend the sensing range.

### 5.8. Threats to Validity

The first threat to validity is the network delay, although in this study, we did not consider communication and network delay. Regarding the network delay, the insights from our study are that it can cause problems in collaborative perception in terms of false detection or introduce more false positives due to the misalignment of frames or missing frames. Network delays should be rigorously investigated as missing frames, or misaligned incoming frames due to network delays can compromise the performance of object detection models. However, we handle this thread to validity by reducing the frame size through compression without losing important features. From the experimental result, we can observe that the average frame size was recorded as only 357 kbit. To process frames with the size of 357 kbit at the rate of 10 frames/s, we need only 3.7 MB/s bandwidth. The commercially available wireless communication system can easily support such a processing rate. 

The second threat to validity is the broad security protocol and data integrity aspects. The article does not cover the broad aspect of the security protocol of data being transferred from one ADS_1 to another ADS_1. Data security and integrity are very important in connected vehicles and need to be thoroughly investigated. Malicious ADSs may send phantom information. Additionally, the participating ADSs in collaborative perception can be unintentionally malicious due to sensor degradation of faulty sensors. This may pose serious driving hazards causing accidents. However, this issue was minimized through the encryption technique. The communication between the two ADSs was secured through an end-to-end encryption technique. On the other hand, if the tampering in image frames happens before the encryption, the encryption systems cannot avoid such tampering, which is another external threat to the validity of this proposed system. Another important aspect is communication with low latency in practical applications. Latency is a big issue that needs to be solved in practical applications. We argue that the advancement in wireless communication has actively accommodated the latency issues. For example, the 5G and millimeter-wave communication provide extremely low latency [44].

Testing safety-threatening scenarios, as shown in Figure 1, with actual vehicles may pose a risk to human safety. Therefore, in general, these risk scenarios must be tested thoroughly by simulated-based testing to ensure safety. Typically, manufacturers perform very limited infield testing to test complex systems such as ADS [45]. Simulation-based testing allows for the safe testing of hazardous scenarios. Hence, manufacturers test more risk scenarios by recording sensor data from infield testing and regenerating them in the simulation environment. Therefore, we decided to validate the proposed system in the simulation environment. We believe that testing the proposed system will yield the same results on real data since the simulator-generated data produces the same results as those obtained on the real data, as mentioned in [46]. However, on-road testing is necessary for the final product to be released, which is done by professional field testers.

## 6. Conclusions

We propose a collaborative perception system to facilitate the ADSs to combine their sensing data with their co-operator to enhance the perceptual ability and situational awareness regarding the occluded objects, thereby improving detection confidence and traffic safety. To the best of our knowledge, no existing works have been reported to implement the concept of the homogeneous sensor (i.e., camera sensor) data fusion of multiple ADSs for collaborative perception.

From experimental results, we conclude that not only does the collaborative perception system increases the detection confidence, but it also increases the object detection rate. The proposed collaborative perception increases the detection rate up to 45.4% more than individual perception. Safety analysis showed that collaborative perception positively impacted on safety of ADSs, as it increased the safety distance up to 1.2 m and the reaction time up to 1 s compared to the individual perception systems. The collaborative perception system detected occluded objects and objects beyond the sensor range through data fusion with nearby ADSs. Additionally, from the experimental results, we confirmed that the proposed collaborative perception system added the benefits of being lightweight, and required low bandwidth communication links, enabling the possibility of being used in practical applications with commercially available low-cost communication links. The comparative analysis of the proposed system with the existing benchmark revealed that our proposed system outperformed existing benchmarks in terms of detection precision and object detection rate. From the experimental results and evaluation, we confirmed that the data volume and time required for transmitting the data from one ADSs to another fall under an acceptable range of commercially available communication links and are feasible for processing the shared data on the local processor of ADSs. We also believe that the proposed system’s computational and communication specifications are feasible for the edge computing environment.

In the future, we aim to investigate more ADSs sensor data fusion for detection and localization where the computation time and bandwidth requirements can be more challenging. We will validate the performance of collaborative perception with more participating ADSs and their effect on communication and processing time. Additionally, with high confidence in the safety of the proposed technique, our future research will focus on conducting on-road testing of the proposed system. We will also investigate the compression technique for transferring data while maintaining the object detection performance.

## Figures and Tables

**Table 1:** The detection performance of individual perception systems of ADS_1, ADS_2 vs. collaborative perception system.

Testing Scenarios	Mean Average Precision (*mAP*)								
	ADS_1			ADS_2			Collaborative Perception System of ADS_1		
	IoU = 0.5	IoU = 0.7	IoU = 0.95	IoU = 0.5	IoU = 0.7	IoU = 0.95	IoU = 0.5	IoU = 0.7	IoU = 0.95
T-junctions	79.1	75.2	30.5	80.2	75.6	31.5	85.4	75.3	35.3
Intersection	78.3	76.1	33.6	79.6	78.3	30.4	84.2	78.8	36.4
Roundabout	78.2	75.6	29.2	77.3	76.1	28.6	78.6	82.3	36.1
Multilane road (opposite direction)	80.1	78.6	27.9	78.6	79.4	28.9	80.9	79.9	29.0
Multilane road (same direction)	79.8	77.3	28.8	80.4	78.2	28.4	86.8	83.2	35.2

**Table 2:** The average communication cost and computation time.

Communication Cost (Kilobit)	Computation Time (Millisecond)	Frame Rate (Frames/s)
357 kbit	301 ms	10 frames/s

**Table 3:** Comparison with existing approaches.

Approaches	Dataset	Sensor Placement	Encryption	Safety Analysis
F-Cooper: feature-based co-operative perception for the autonomous vehicle using 3D point clouds [10]	LiDAR Point Clouds	Mounted on ADSs	**✕**	**✕**
Cooper: co-operative perception for connected and autonomous vehicles based on raw data. [32]	LiDAR Point Clouds	Mounted on ADSs	**✕**	**✕**
Co-operative perception using infrastructural sensors. [33]	LiDAR Point Clouds	Fixed Location	**✕**	**✕**
**This work**	**Camera Image**	**Mounted on ADSs**	**✓**	**✓**
